# Electrical properties based $B_{1}^{+}$ prediction for electrical properties tomography reconstruction evaluation

**DOI:** 10.1002/mrm.30520

**Published:** 2025-04-02

**Authors:** Thierry G. Meerbothe, Kyu‐Jin Jung, Chuanjiang Cui, Dong‐Hyun Kim, Cornelis A. T. van den Berg, Stefano Mandija

**Affiliations:** ^1^ Department of Radiotherapy, Division of Imaging and Oncology University Medical Center Utrecht Utrecht The Netherlands; ^2^ Computational Imaging Group for MR Therapy and Diagnostics, Center for Image Sciences University Medical Center Utrecht Utrecht The Netherlands; ^3^ Department of Electrical and Electronic Engineering Yonsei University Seoul Republic of Korea

**Keywords:** conductivity, confidence, electrical properties tomography, finite differences

## Abstract

**Purpose:**

In MR electrical properties tomography (EPT), conductivity and permittivity are reconstructed from MR measurements. However, depending on the reconstruction method, reconstructed electrical properties (EPs) show large variability in vivo, reducing confidence in the reconstructed values for clinical application in practice. To overcome this problem we present a method to evaluate the reconstructed EPs using a physics‐based $B_{1}^{+}$ estimation model.

**Methods:**

A physics‐based method using a finite difference based recurrent relation is used to estimate the $B_{1}^{+}$ field from a set of given EPs and the boundary of the measured $B_{1}^{+}$ field. Reconstructed EPs can be evaluated by comparing the estimated $B_{1}^{+}$ field with the measured $B_{1}^{+}$ field. The method was first validated in simulations and afterward tested using MRI data from phantoms and in vivo.

**Results:**

The simulation experiments show that the $B_{1}^{+}$ field can be accurately estimated, within 90 s for a typical brain at 1 mm^3^ isotropic resolution, when correct EPs are used as input. When incorrect EPs are used as input the estimated $B_{1}^{+}$ fields shows differences with the measured $B_{1}^{+}$ fields. These differences directly correspond to the errors in the underlying EPs, enabling detection of errors in the reconstructions. The results obtained in MRI experiments using phantoms and in vivo show the applicability of the method in practice.

**Conclusion:**

With the proposed method, $B_{1}^{+}$ fields can be accurately estimated from EPs. This approach can be used to evaluate EPT reconstructions and consequently gain more confidence in reconstructed EPs values in vivo.

## 1 INTRODUCTION

Electrical properties tomography (EPT) is a technique that aims to reconstruct electrical properties (EPs, conductivity σ and relative permittivity ε_r_) from MRI measurements of the transmit $B_{1}^{+}$ field. At MRI frequencies (MHz range), EPs of biological tissues are mainly dependent on the interaction between electromagnetic fields and the ionic composition of tissue.^1^, ^2^ Because this composition is highly varying in biological tissue, EPs throughout the human body are also very heterogeneous.^3^ Consequently, EPs could act as potential biomarker in several clinical applications,^3^ which has fueled recent interest in research on EPT.

An example of clinical application of EPT is the identification and characterization of tumors in oncology. Studies have shown significantly higher conductivity in brain and breast tumors compared to healthy tissue,^4^ which can be measured with EPT.^5^, ^6^, ^7^, ^8^, ^9^ Additionally, it was suggested that different tumor components can be identified using EPT.^10^, ^11^ Other oncological applications such as the assessment of tissue response after radiotherapy have also been proposed.^12^, ^13^ Currently, even applications outside of oncology have been investigated including conductivity‐based fMRI^14^, ^15^ and stroke detection.^16^

For clinical application, accurate and consistent reconstruction of the EPs is necessary. EPs can be reconstructed from the measured, complex transmit $B_{1}^{+}$ field.^3^, ^17^ However, often several assumptions are used to reconstruct the EPs, such as the local homogeneity assumption and the phase‐only assumption. This can cause errors in the reconstructions such as artifacts at tissue boundaries.^18^, ^19^ Additionally, the calculation of spatial derivatives of the measured $B_{1}^{+}$ field is heavily influenced by noise. Despite the first promising clinical evidences on the use of EPs as biomarkers, accurate reconstructions of EPs have proven difficult in practice as also shown by the large variation in in vivo reconstructed EPs using the widely used Helmholtz reconstruction^20^ and the initial results of the EPT reconstruction challenge.^21^

More recently, deep learning (DL)‐based reconstruction methods have emerged, which overcome a lot of the problems of conventional, physics‐based EPT reconstructions.^22^, ^23^, ^24^, ^25^ However, “black‐box” DL‐based reconstruction methods come with their own set of problems. For training, accurate (ground truth [GT]) EPs are necessary. Because these are not available in vivo, training is typically done using simulated data.^26^ Therefore, it is uncertain how the reconstructions generalize to in vivo data or to out of distribution pathological cases.^23^ Recently, physics‐informed neural networks have been explored to alleviate such generalization problems.^27^, ^28^, ^29^, ^30^ However, trust on the quality of reconstructed EP maps in vivo with DL remains an open question.

The large variability in reconstructions from different reconstruction methods,^21^ the large inter‐subject variability in the reported in vivo EPs, and the uncertainty in DL‐based reconstructions, limits the confidence on reconstructed EPs in vivo. This makes clinical application of EPT reconstructions, and in particular DL‐based reconstructions, difficult in practice. To address the lack of trust on the reconstructed EP maps, we developed a fast, physics‐based method to provide confidence on EPT reconstructions. The proposed method uses a recurrent finite difference (FD) formulation to estimate complex $B_{1}^{+}$ fields from EPs maps reconstructed using an arbitrary EPT method. By comparing the estimated and measured $B_{1}^{+}$ fields, it is possible to evaluate the reconstructed EP maps and identify regions where inaccurate EPs have been reconstructed. The ability to evaluate reconstructed EPs can alleviate uncertainties related to DL‐based reconstructions and can provide confidence to in vivo reconstructed EPs, whereas the speed and simple setup of the method makes it applicable in clinical practice. This will help clinical application of EPT in the future. Because EPT is mainly focused on conductivity estimation,^21^ this work mainly focuses on the evaluation of conductivity using $B_{1}^{+}$ estimation.

## 2 THEORY

### 2.1 Reconstruction of EPs from $B_{1}^{+}$ field: EPT governing equations

The complex transmit field $B_{1}^{+}=|B_{1}^{+}|e^{i\phi^{+}}$ (|$B_{1}^{+}$| = transmit magnitude and *ϕ* ^+^ = transmit phase) and the EPs are related according to^31^:

(1) $$\begin{array}{ll} & -\nabla^{2}B_{1}^{+}=\omega^{2}\mu_{0}\varepsilon_{c}B_{1}^{+}-\left(g_{x}+ig_{y}\right)\left(\frac{\partial B_{1}^{+}}{\partial x}-i\frac{\partial B_{1}^{+}}{\partial y}+\frac{1}{2}\frac{{\partial B}_{z}}{\partial z}\right)\\ & \;-g_{z}\left(\frac{\partial B_{1}^{+}}{\partial z}-\frac{1}{2}\frac{{\partial B}_{z}}{\partial x}-i\frac{1}{2}\frac{{\partial B}_{z}}{\partial y}\right), \end{array}$$

where ω = Larmor frequency, μ_0_ = vacuum permeability, $\left(g_{x},g_{y},g_{z}\right)=\nabla \ln\left(\varepsilon_{c}\right)$, with ε_*c*_ = complex permittivity (ε_*0*_ε_r_‐i*σ/ω*), consisting of the relative permittivity ε_*r*_, with ε_0_ the vacuum permittivity, and conductivity σ. Under the assumption that ∇B_z_ is negligible, which generally holds in the center of a birdcage coil,^32^ Eq. [1] can be simplified as:

(2) $$-\nabla^{2}B_{1}^{+}=\omega^{2}\mu_{0}\varepsilon_{c}B_{1}^{+}-\left(g_{x}+ig_{y}\right)\left(\frac{\partial B_{1}^{+}}{\partial x}-i\frac{\partial B_{1}^{+}}{\partial y}\right)-g_{z}\frac{\partial B_{1}^{+}}{\partial z}.$$

This complex valued partial differential equation can be further simplified by assuming EPs to be piecewise constant (*g*_*x,y,z*_ = 0). This leads to the so called Helmholtz equation, which is the cornerstone equation for EPT reconstructions:

(3) $$\frac{\nabla^{2}B_{1}^{+}}{B_{1}^{+}}=-\omega^{2}\mu_{0}\varepsilon_{c}.$$

Equation [3] can be simplified further, relating ϕ^+^ to conductivity and |$B_{1}^{+}$| to permittivity in the so called phase‐only and magnitude‐only EPT approximations^17^:

$$\nabla^{2}\phi^{+}=\omega \mu_{0}\sigma$$

(4) $$\frac{\nabla^{2}|B_{1}^{+}|}{\left|B_{1}^{+}\right|}=-\omega^{2}\mu_{0}\varepsilon_{0}\varepsilon_{r}.$$

### 2.2 Reconstruction of $B_{1}^{+}$ field from EPs

Instead of using Eq. [1] to calculate the EPs, in this work the forward problem is solved, calculating the $B_{1}^{+}$ field from a map of reconstructed EPs, independently from the EPT method used to reconstruct them. The calculated $B_{1}^{+}$ field is then used to evaluate the accuracy of reconstructed EPs as described in Section 3 .

To solve for $B_{1}^{+}$, Eq. [1] can be posed as boundary value problem in an arbitrary domain Ω with boundary ∂Ω. The EPs in Ω are considered known. On ∂Ω, Dirichlet boundary conditions are set for $B_{1}^{+}$ equal to the measured $B_{1}^{+}$. To solve this problem, we use a simple iterative relaxation method, resulting in a recurrent expression based on central FDs, derived from Eq. [1] (Appendix A1). This expresses the $B_{1}^{+}$ in terms of the $B_{1}^{+}$ and *B*_*z*_ in neighboring voxels and the corresponding EPs for all *N* voxels in Ω^33^:

(5) $$B_{i+1}=\frac{AB_{i}+A_{z}Z}{6-\omega^{2}\mu_{0}Eh^{2}},$$

where *B* ([*N* × 1]) represents the $B_{1}^{+}$ field, *Z* ([*N* × 1]) contains the *B*_*z*_ field, and *E* ([*N* × 1]) is the complex permittivity. *A* and *A*_*z*_ are FD‐based operators (sparse matrices [*N* × *N*]) acting on the off‐diagonal terms of *B* and *Z*, respectively (See Appendix A1).^34^ Finally, *h* denotes the resolution and *i* the iteration number.

**FIGURE 1:**
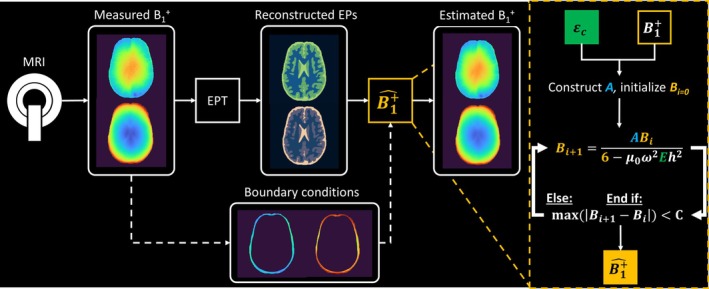
Schematic pipeline for the prediction of $B_{1}^{+}$ in an MRI experiment. With a measured $B_{1}^{+}$ field from MRI, an arbitrary electrical properties tomography (EPT) reconstruction is done to retrieve electrical properties (EPs). Using theseEPs and the measured $B_{1}^{+}$ field at the boundary of the region of interest, a $B_{1}^{+}$ field can be estimated, as summarized on the right.

This formulation can be used to estimate the $B_{1}^{+}$ in an arbitrary domain by constraining $B_{1}^{+}$ on the outer voxel layer (boundary ∂Ω) of Ω to be equal to the measured $B_{1}^{+}$ field. Additionally, homogeneous Neumann boundary conditions are imposed for ε_*c*_ (∇ε_*c*_ = 0), such that no knowledge of the EPs on the boundary is required. Equation [5] is then recurrently applied until $B_{1}^{+}$ between subsequent iterations converges, defined by a limit *C* (max (|*B*_*i+*1_ *−B*_*i*_|) < C). Using a more stable limit (e.g., 90th percentile) had no significant impact on the results. As such, the $B_{1}^{+}$ field can be estimated. For application within an MRI experiment, a schematic pipeline of $B_{1}^{+}$ prediction with this method is shown in Figure 1.

The formulation in Eq. [5] requires knowledge of *B*_*z*_, which is not measureable in practice. When taking the assumption of negligible ∇*B*_*z*_ ^31^, ^35^ (corresponding to the simplification of Eqs. [1–2]), the recurrent formulation is simplified into:

(6) $$B_{i+1}=\frac{AB_{i}}{6-\mu_{0}\omega^{2}Eh^{2}}.$$

This can be used to estimate $B_{1}^{+}$ in a realistic scenario without any knowledge of *B*_*z*_.

In addition to *B*_*z*_, the transmit phase (ϕ^+^) is also not directly measureable in an MRI experiment, as the measured RF phase is affected by both the transmit and receive phase (ϕ^−^)^36^:

(7) $$\phi^{\pm }=\phi^{+}+\phi^{-}.$$

Hence, in MR‐EPT, ϕ^+^ is usually approximated as half of the measured transceive phase (ϕ^±^) (transceive phase assumption), which is a valid approximation at field strengths of 3 T and below.^36^

Because only ϕ^±^ is measureable in an actual MRI experiment, $B_{1}^{+}$ predictions will consequently be done using ϕ^±^ as boundary condition. The error that results from this is limited, as shown in Figure S1. Throughout this work the transceive phase assumption is, therefore, used to approximate ϕ^+^ and is consequently used in the $B_{1}^{+}$ calculation.

## 3 METHODS

Complex $B_{1}^{+}$ field predictions were performed both on simulated and measured data.

### 3.1 $B_{1}^{+}$ prediction experiments on simulated data

Simulated data were created by performing electromagnetic simulations in Sim4Life (ZMT) using a quadrature driven birdcage coil tuned at 128 MHz (3 T).^26^ Three models were used in these simulations: (1) A spherical phantom with radius 80 mm (σ = 0.8 S/m, ε_*r*_ = 70) containing an off‐center inner spherical compartment with radius 40 mm (EPs σ = 0.3 S/m, ε_*r*_ = 60), Ω ≈ 2.1e^6^ voxels. (2) A simulated brain model from the ADEPT database, with conductivity 0.35, 0.69, and 2.26 S/m and permittivity 51, 73, and 80 for white matter (WM), gray matter (GM), and CSF, respectively.^26^ (3) A brain model with the same geometry as simulated brain model (2), but different EPs (σ = 0.36, 0.68, 2.06 S/m, and ε_*r*_ = 54, 72, 86 for WM, GM, and CSF, respectively). An additional simulation with model (3) was done with a tumor inclusion. For all brain models, Ω ≈ 1.7e^6^ voxels. The tumor consisted of three compartments with EPs: σ = 0.7, 0.9, 1.2 S/m, and ε_*r*_ = 60, 70, and 80. This resulted in a set of simulated *B* fields corresponding to the input (GT) EPs of the different models. In experiments with noise, Gaussian noise was added on |$B_{1}^{+}$| and ϕ^+^ separately. For |$B_{1}^{+}$|, noise was added on the real and imaginary parts of the complex $B_{1}^{+}$ field. For ϕ^+^ noise was added to the real and imaginary parts of a complex field consisting of a synthetic T_1_w magnitude and ϕ^+^ as phase. The resulting SNR was 50 for |$B_{1}^{+}$| and 80 for the synthetic T_1_w image (for ϕ^+^). This emulates noise of a real MRI experiment.^21^

For these models, the outer voxel layer of the simulated volume was used as boundary. The simulated $B_{1}^{+}$ field in these voxels was used as Dirichlet boundary condition. Using this together with the EPs, a $B_{1}^{+}$ field was predicted with Eqs. [5] and [6] in four experiments. The predicted field was afterward compared to the simulated field used as GT. To validate that the model estimates accurate $B_{1}^{+}$ fields, this was first done using the GT EPs as input (experiments 1 and 2 of Table 1). Afterward, the impact of errors in the input EP maps on the predicted $B_{1}^{+}$ field was investigated by modifying input conductivity on purpose (experiment 3 [sensitivity in a simple model] and experiment 4 [sensitivity in a realistic case] of Table 1). In all experiments, |$B_{1}^{+}$| was normalized to the maximum value, the convergence limit C was set to 1e−6, and the experiments were initialized with a uniform $B_{1}^{+}$ map of value 1, for magnitude and phase.

**TABLE 1:** Overview of the four simulation experiments done to validate the proposed model and to test effect of imperfect input EPs.

Simulation experiments		Phantom	∇*B*_*z*_ = 0	Noise	GT EP input	Erroneous input *σ*
Model validation	1	Brain	×	×	✓	n.a.
	2	Sphere + brain	✓	SNR50/80	✓	n.a.
Impact of erroneous input σ on predicted $B_{1}^{+}$ field	3	Sphere	✓	SNR50/80	×	‐ σ offset (± 0.05 S/m) ‐ Inner compartment size change (± 2 voxels) ‐ Anomaly (0.2 S/m, 0.6 S/m)
	4	Tumor brain	✓	SNR50/80	×	‐ Tumor removed from input

Abbreviations: EPs, electrical properties; GT, ground truth.

### 3.2 $B_{1}^{+}$ prediction experiments on phantom MRI data

Next, we tested the applicability and sensitivity of the proposed method in MRI measurements on phantoms. For this purpose, three spherical phantoms were constructed (diameter 12 cm), with different saline gelatin solutions (σ_1_ = 0.41, σ_2_ = 0.51, σ_3_ = 0.61 S/m^37^). All phantoms had a relative permittivity of 78 (permittivity of water).

The phantoms were scanned using a clinical 3 T Ingenia CX MRI system (Philips Healthcare). The body coil was used in transmit mode and a 15‐channel head coil was used for receive mode. Additionally, the vendor CLEAR function was used to correct for receive coil inhomogeneities.^19^ Transceive phase images were sagitally acquired using two 3D balanced steady state free precession (bSSFP) sequences with opposite readout polarity, averaged afterward (1 mm^3^ isotropic resolution, FOV = 192 × 192 × 192 mm^3^, TR = 3.02 ms, TE = 1.51 ms, flip angle (FA) = 30°).^23^ The phase maps were divided by two to get the approximated transmit phase.^36^ $B_{1}^{+}$ magnitude data was acquired using a dual refocusing echo acquisition mode acquisition (resolution 3 × 3 × 3 mm^3^, FOV = 192 × 192 × 192 mm^3^, *T*_*R*_ = 5.21, *T*_E1_ = 1.93 ms, *T*_E2_ = 2.6 ms, FA = 20°).^38^ The |$B_{1}^{+}$| data was reconstructed on a 1 × 1 × 3 mm^3^ grid on the scanner and afterward linearly interpolated in the slice direction to match the 1 mm isotropic resolution of the transceive phase map. For all phantoms Ω ≈ 0.8e^6^ voxels.

Three experiments were done with these phantoms:

(1) Phantom experiment 1: the applicability of the method was tested on measured data. For each sphere, $B_{1}^{+}$ estimation was done three times. Once using the correct reference EPs as input and twice using the input EPs of the two other spheres.

(2) Phantom experiment 2: the applicability of $B_{1}^{+}$ estimation based on EPT reconstructions was tested on phantom 3 (σ_3_ = 0.61 S/m). Conductivity was reconstructed using phase‐based EPT (Eq. [4]) and complex Helmholtz EPT (Eq. [3]). Both were reconstructed using a 3‐point derivative kernel and the result was denoised using a spherically shaped 11‐point mean filter. These reconstructed conductivity maps (together with water permittivity) were used as input for $B_{1}^{+}$ reconstruction.

(3) Phantom experiment 3: the model sensitivity to anomalies in the input EPs was tested by including an artificial anomaly in the input conductivity map. The anomalies were varied in size (9–21 mm) and conductivity offset (0.2–0.8 S/m) with respect to the reference conductivity. Furthermore, the anomalies were placed in two different locations of the phantom to assess the impact of the location on reconstruction errors: In the center and on the periphery.

### 3.3 $B_{1}^{+}$ prediction experiments on in vivo MRI data

Finally, the method was applied to an in vivo brain scan of a healthy volunteer previously acquired for the EPT reconstruction challenge.^21^ The reuse of in vivo imaging data was in agreement with the study approved by the institutional review board of the UMC Utrecht. Here, transceive phase images were again acquired using a 3D bSSFP sequence (1 mm^3^ isotropic resolution, FOV = 224 × 224 × 224 mm^3^, TR = 3.8 ms, TE = 1.88 ms, FA = 30°, number of signal averages = 2).^22^ The transmit phase was approximated by half the transceive phase. $B_{1}^{+}$ magnitude data was acquired using an actual flip‐angle imaging acquisition (resolution 4 × 4 × 3 mm^3^, FOV = 256 × 192 × 180 mm^3^, TR_1_ = 50 ms, TR_2_ = 250 ms, TE = 2.3 ms, FA = 65°).^39^ It was linearly regridded to 1 mm^3^ isotropic resolution, matching the resolution of the transceive phase. Here, the region of interest (ROI) is an eroded brain mask, Ω ≈ 1.3e^6^ voxels.

With this in vivo data two things were tested: (1) the applicability of $B_{1}^{+}$ prediction to in vivo data and (2) the sensitivity to a synthetic anomaly in the input EPs (false positives in reconstruction), similarly to phantom experiment 3.

As input, an EPs were assigned based on a brain segmentation of WM, GM, and CSF created by thresholds based on the bSSFP signal magnitude. Each tissue was then assigned a single conductivity and permittivity value: 0.36, 0.67, and 2.14 S/m for conductivity and 54.5, 65, and 78 for permittivity, respectively. As synthetic anomaly, conductivity was changed within an elliptical region (10–15 mm radius, σ = 0.9 S/m) with a smaller spherical compartment (5 mm radius, σ = 1.3 S/m). Permittivity remained equal to the value assigned after the segmentation.

### 3.4 Application of $B_{1}^{+}$ prediction for EPT reconstruction evaluation

The predicted $B_{1}^{+}$ fields are directly dependent on the EP map used as input. Therefore, the difference between the estimated $B_{1}^{+}$ and the reference (measured) $B_{1}^{+}$ field can be used as an indication of the accuracy of the reconstructed EPs. The difference between the $B_{1}^{+}$ maps are given by:

$$D_{\phi }=∠\hat{(B_{1}^{+}}(\varepsilon_{c}))-\phi^{+}$$

(8) $$D_{B}=|\hat{B_{1}^{+}}\left(\varepsilon_{c}\right)|-|B_{1}^{+}|,$$

where the hat denotes the estimated $B_{1}^{+}$ field using the recurrent model and is compared to the measured, noisy reference maps. These *D_ϕ_* and *D*_*B*_ difference maps show how well the estimated and measured fields match overall. However, local differences in EPs affect the field globally. To locally identify errors in the EPs, the linearity of magnitude and phase‐based EPT relations in Eq. [4] are used: because |$B_{1}^{+}$| and ϕ^+^ are related to the permittivity and conductivity with the second order derivative, the difference of the fields is also related to a difference in the EPs. Therefore, to localize errors in the input EPs, surrogate conductivity and permittivity error maps *L*_ϕ_/*L*_*B*_ are created by calculating second order derivatives:

$$L_{\phi }=\frac{\nabla^{2}\left(S\left(D_{\phi }\right)\right)}{\omega \mu_{0}}$$

(9) $$L_{B}=\frac{\nabla^{2}\left(S\left(D_{B}\right)\right)}{\omega^{2}\mu_{0}\varepsilon_{0}},$$

here, *S* denotes a smoothing function needed to suppress noise artifacts. For this, a Gaussian smoothing function was used with standard deviation of 4 in an 11‐voxel kernel.

The predicted $B_{1}^{+}$ fields from simulation experiments 1–2, where GT EPs are used as input, were evaluated using the difference maps *D* and the mean absolute error (MAE) of *D*. The other experiments, where erroneous EPs are present in input, were additionally evaluated using surrogate EP error maps *L*. Finally, the median of the *L*_ϕ_ map was calculated, as an indirect measure of the corresponding global offset in the input conductivity and is indicated by *L*_ϕ_^off^.

Although the complex permittivity was used for $B_{1}^{+}$ field prediction in all experiments, only the estimated phase maps were analyzed from experiment 3 onward for clarity. Equal analysis can, however, be applied to the estimated |$B_{1}^{+}$| maps, where effects of mainly the permittivity would then be studied. Furthermore, for erroneous input, only conductivity was changed in input, because this is the main focus of this work. The permittivity was kept the same.

## 4 RESULTS

### 4.1 Model validation and EP evaluation using simulated data

**FIGURE 2:**
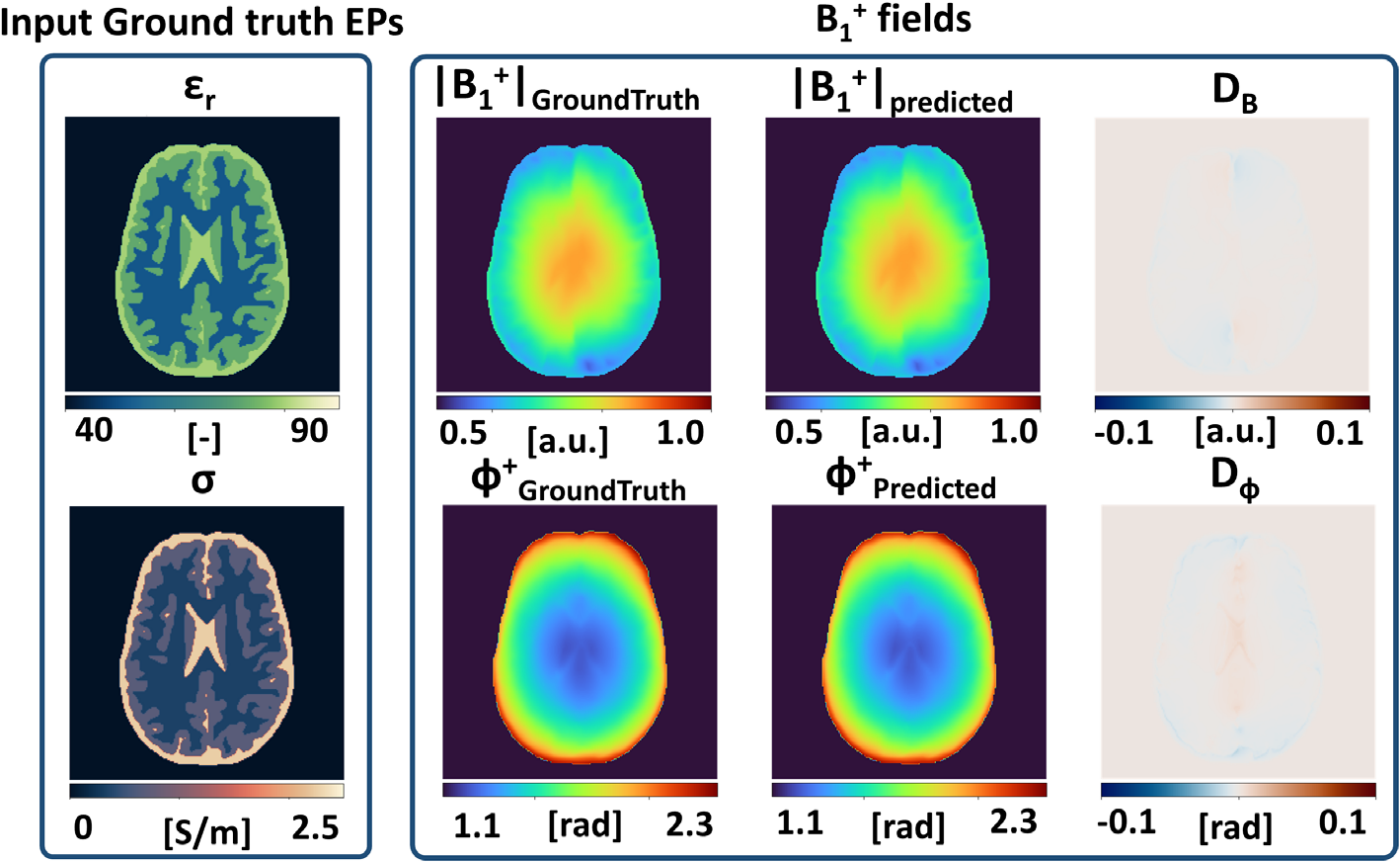
Calculated $B_{1}^{+}$ field and difference maps for $B_{1}^{+}$ prediction including the field component B_z_ in a noiseless simulated brain model. Ground truth electrical properties (EPs) were used as input. |$B_{1}^{+}$| has been normalized to the maximum value.

Simulation experiment 1: the results of estimation of $B_{1}^{+}$ using Eq. [5] (including *B*_*z*_) for the simulated brain model are shown in Figure 2 for a central axial slice. For this brain model, (1.5 million voxels with 1 mm^3^ isotropic resolution) estimation took approximately 1.5 min using a Nvidia GeForce RTX 3050 GPU for acceleration. This can be further decreased to 50 s by initializing the method with a non‐uniform, analytically estimated $B_{1}^{+}$ map or to 30 s using the measured $B_{1}^{+}$ as input.

The predicted $B_{1}^{+}$ fields match the simulated reference fields (GT) with MAE = 2.6e−3 and 1.6e−3 rad for the normalized |$B_{1}^{+}$| and ϕ^+^, respectively, over the full volume. Different orientations (sagittal and coronal) show similar results (see Figures S2 and S3).

**FIGURE 3:**
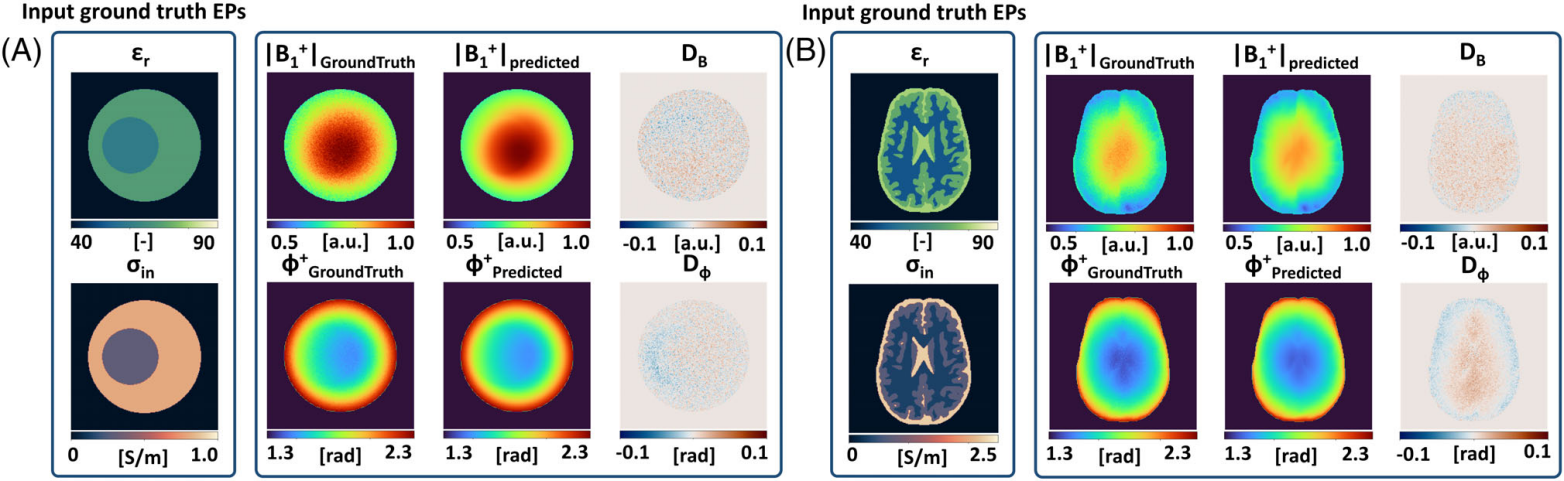
Calculated $B_{1}^{+}$ field and difference maps for $B_{1}^{+}$ prediction excluding the field component *B*_*Z*_ in a spherical phantom with two compartments (A) and a simulated brain (B) in a noisy (SNR = 50/80) case. Ground truth electrical properties (EPs) were used as input.

Simulation experiment 2: the results on the sphere phantom and simulated brain phantom with the assumption ∇*B*_*z*_ = 0 are shown in Figure 3 in presence of noise.

For the sphere phantom (Figure 3A), the predicted |$B_{1}^{+}$| and ϕ^+^, match the simulated reference with MAE = 1.3e−2 for |$B_{1}^{+}$| and 1.0e−2 rad for ϕ^+^ over the full volume. The predicted $B_{1}^{+}$ maps are robust to noise in the reference $B_{1}^{+}$ map, because the noisy $B_{1}^{+}$ data is only used as boundary condition. A small error of up to 2e−2 rad can be observed around the interface of the two compartments in the phase map. This is a direct result of the exclusion of *B*_*z*_.

For the brain phantom (Figure 3B), slightly higher errors are present with MAE = 1.4e−2 for |$B_{1}^{+}$| and 1.7e−2 rad for ϕ^+^. Although differences are still low, they are higher in comparison to the model without assumptions on *B*_*z*_ (Figure 2). This can be explained by the more intricate geometry with more inhomogeneous EPs (nonzero *g*_*x,y,z*_). Consequently larger errors because of the assumption ∇*B*_*z*_ = 0 arise. For the brain model, similar results are shown in sagittal and coronal view (Figures S4 and S5).

**FIGURE 4:**
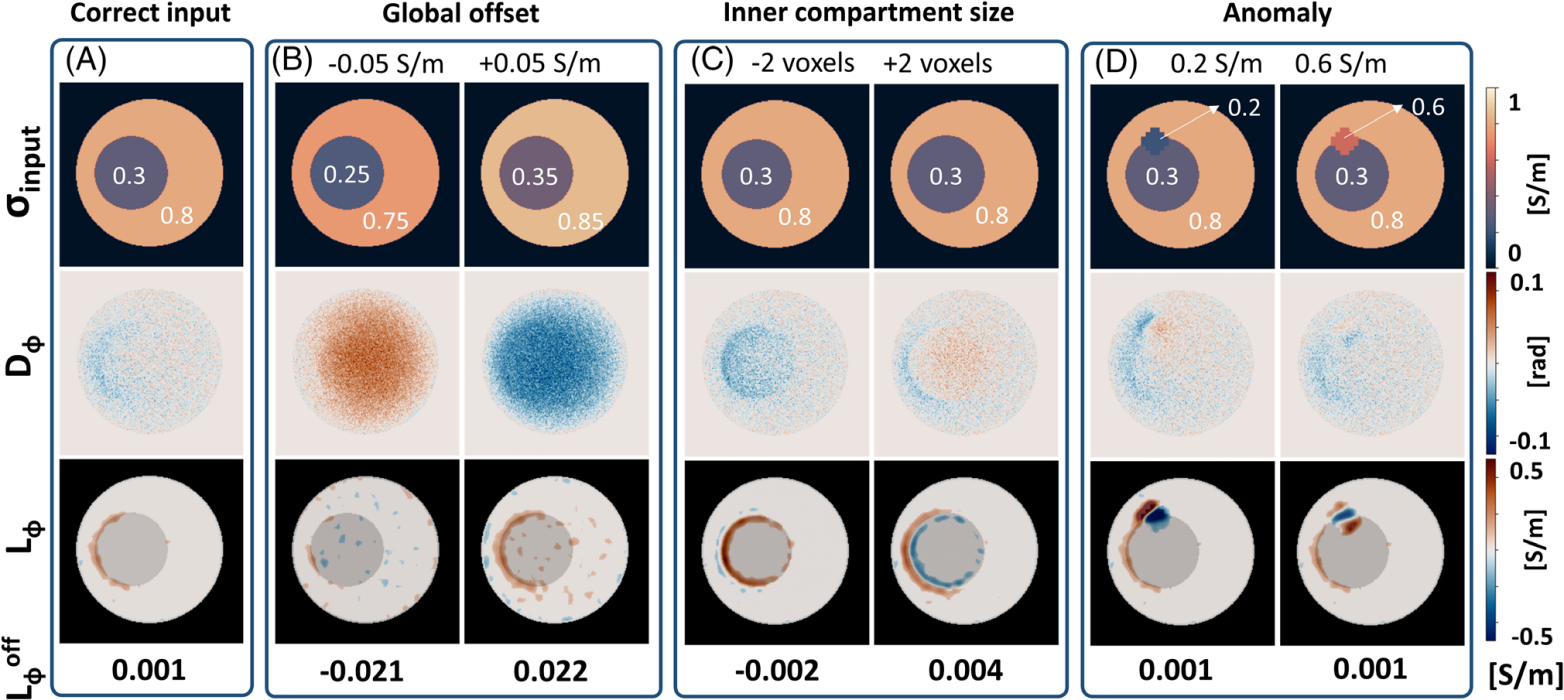
Difference maps (*D*_ϕ_) and error maps (*L*_ϕ_) from $B_{1}^{+}$ prediction in a single spherical phantom, using different input conductivities. The *L*_ϕ_ maps are masked to only show values >0.1 S/m, with which effects of noise are excluded.

Simulation experiment 3: the results from using erroneous input conductivity in the spherical phantom are shown in Figure 4. Figure 4(A) shows *D*_ϕ_ and *L*_ϕ_ maps for the reference case with perfect input EPs. The results in (A) are equivalent to Figure 3, including the model‐related error because of the assumption on *B*_*z*_. This is consequently also present in all other results.

The *D*_ϕ_ maps clearly show larger errors when erroneous conductivity values are used in input (Figure 4B–D). The largest error (MAE = 2e−2 rad) is visible for a global offset ±0.05 S/m (Figure 4B). Here, no clear errors are shown in *L*_ϕ_ because the applied offset in input conductivity is smaller than the cutoff value of the *L*_ϕ_ maps of 0.1 S/m. However, the offset is reflected by the *L*_ϕ_^off^ value.

Changing the size of the inner compartment in the input conductivity in Figure 4C results in a larger difference *D*_ϕ_ within the inner sphere. The *L*_ϕ_ map clearly reflects the local conductivity error, located at the rim of the inner compartment. Finally, when adding an anomaly in Figure 4D, big local errors around the anomaly can be observed in the *L*_ϕ_ map. Both have low *L*_ϕ_^off^ values, as no offset in conductivity is present.

**FIGURE 5:**
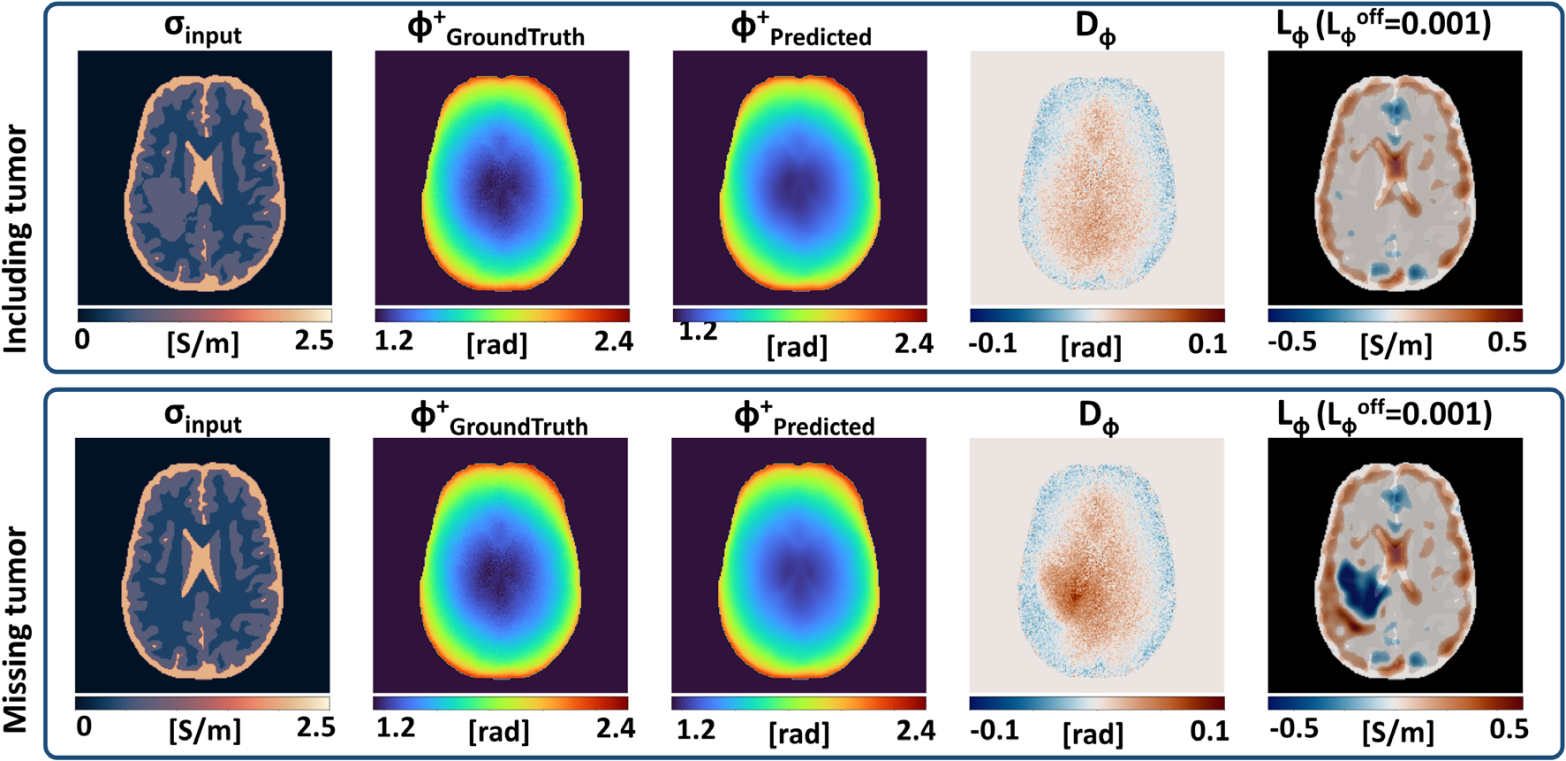
Calculated ϕ^+^ map and corresponding error maps for a simulated brain model with tumor inclusion. Conductivity input in the top row includes the tumor in input, whereas conductivity input in the bottom row incorrectly misses the tumor.

Simulation experiment 4: the results for the brain model with tumor are shown in Figure 5. When the tumor is correctly included in the input EP maps (top row), a similar error is observed as in Figure 3. As result of the assumption ∇*B*_*z*_ = 0, *L*_ϕ_ mostly shows in the CSF and ventricle regions, because of the high EP difference. When the tumor is not reconstructed (bottom row), clear errors arise in the tumor region, indicating that the input conductivity does not match the actual conductivity in that region.

### 4.2 Applicability and sensitivity of EP evaluation on phantom MRI experiments

**FIGURE 6:**
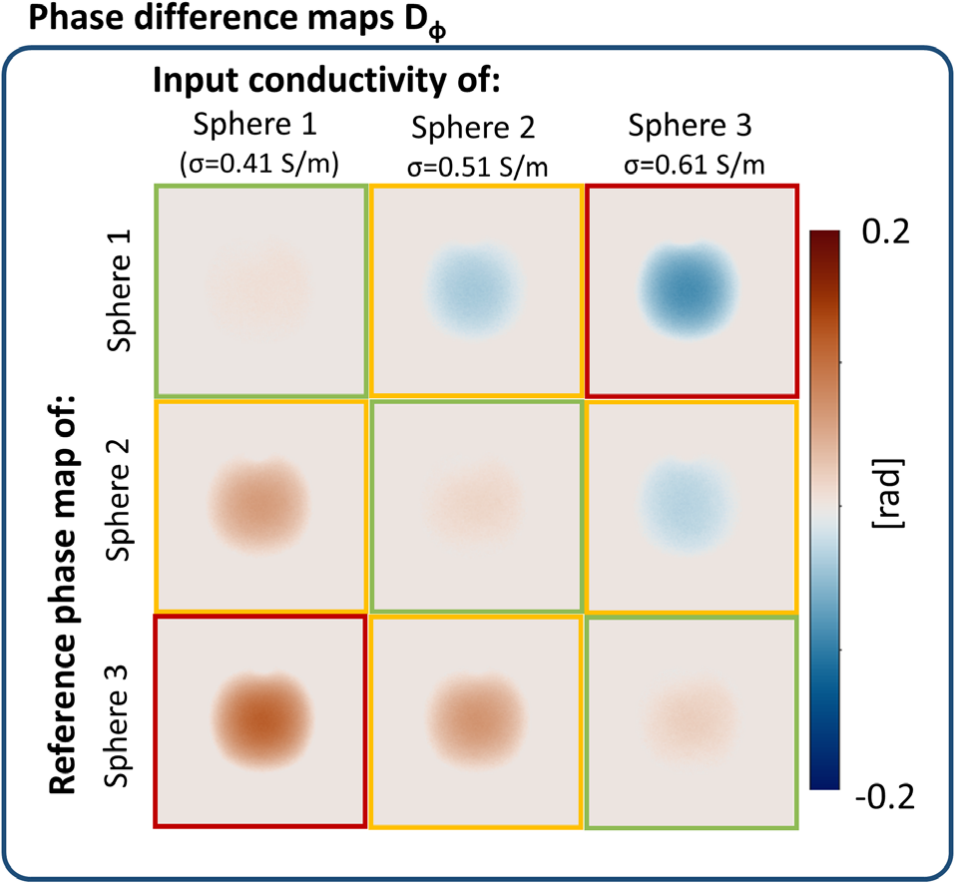
Calculated phase difference maps for three spherical phantoms with different conductivity. Accurate phase maps are predicted when the correct pair of input reference conductivity and measured phase is used, which is indicated with the green boxes (on diagonal). Offset of input conductivity values of ±0.1 S/m are indicated with the yellow border, offsets of ±0.2 S/m are indicated with the red border.

Phantom experiment 1: the results of experiments with the three spherical phantoms are shown in Figure 6. The difference maps *D*_ϕ_ are shown for all combinations of $B_{1}^{+}$ predictions. When the reference conductivity is used as input for the model (green diagonal), *D*_ϕ_ is low with a MAE = 3.0e−3, 5.4e−3, and 7.0e−3 rad for sphere 1, 2, and 3, respectively, which is lower than in the simulated case (Figure 3). Instead, when erroneous conductivity values are used in input (off diagonals in Figure 6), larger differences are present. For an input error of ±0.1 S/m (yellow border), MAE >1.3e−2 rad. For an input error of ±0.2 S/m (red border), MAE >3.4e−2 rad. The proposed method can, therefore, distinguish variations in conductivity values below 0.1 S/m on measured data.

**FIGURE 7:**
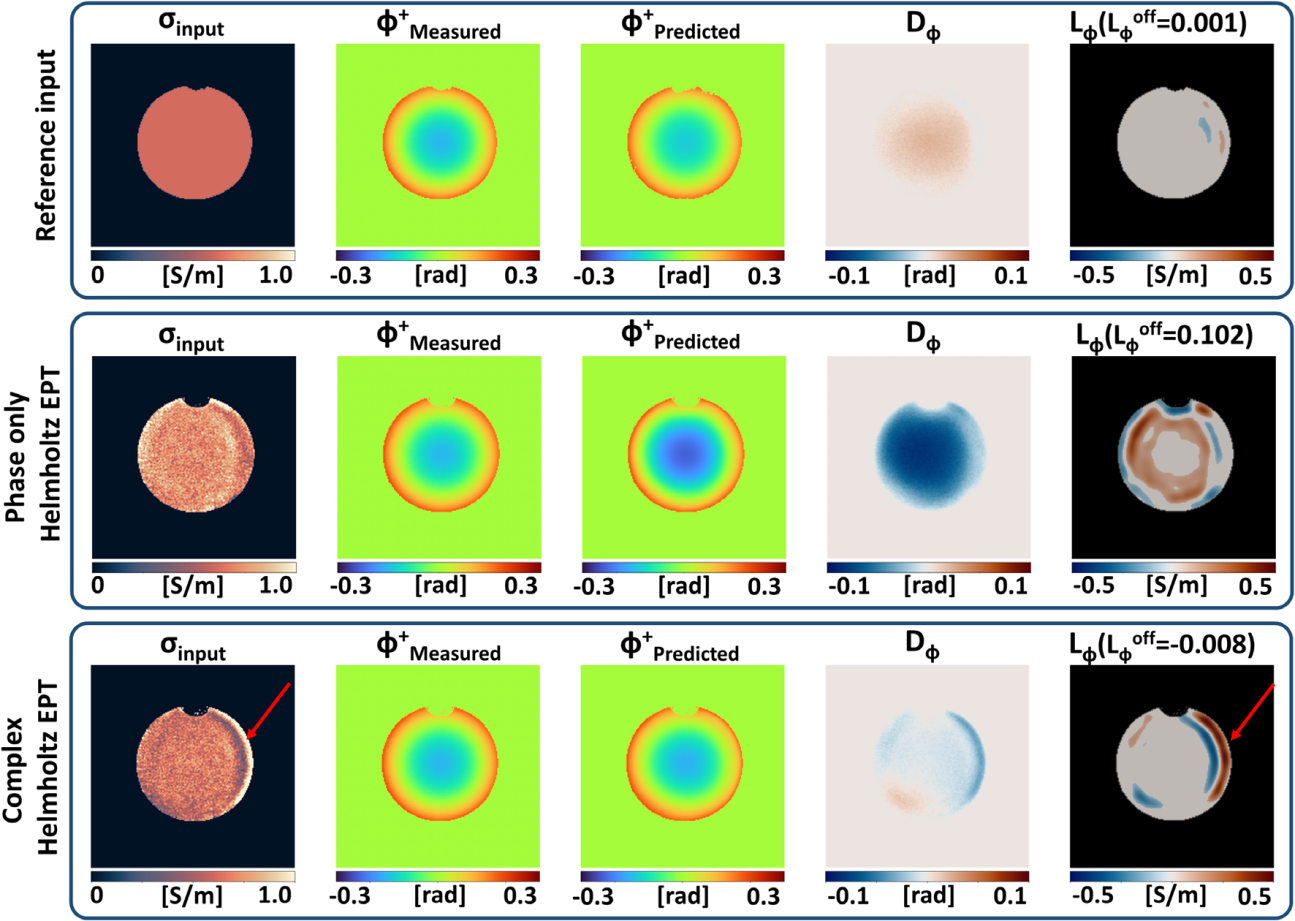
Evaluation of Helmholtz‐based electrical properties tomography (EPT) reconstructions on a spherical phantom. Top row shows the evaluation using reference electrical properties (EPs) as input. Second row uses conductivity maps obtained from phase‐only EPT (Eq. [4]) as input. Third row uses conductivity maps from Helmholtz EPT (Eq. [3]) as input. Red arrows indicate a reconstruction error as a result from a measurement artifact.

Phantom experiment 2: the results from $B_{1}^{+}$ predictions using EPT reconstruction to obtain the input EPs are shown in Figure 7. With the reference conductivity value (0.61 S/m) as input (top row), the results are the same as for sphere 3 in Figure 6. When using conductivity reconstructed with phase‐based EPT (Eq. [4], σ_mean_ = 0.81 S/m) as input (middle row), a much higher discrepancy is shown in *D*_ϕ_ (MAE = 4.7e−2) and *L*_ϕ_. This error is expected because it reflects the known overestimation in *σ* with phase‐based EPT reconstructions.^40^ It is also reflected by the high *L*_ϕ_^off^ value (0.10 S/m). When the conductivity from full Helmholtz reconstruction (Eq. [3], σ_mean_ = 0.67 S/m) is used (bottom row), lower errors are observed in *D*_ϕ_ (MAE = 1.1e−2) and *L*_ϕ_, which is in line with the small difference between the estimated and reference conductivity. As a result of an artifact in the measured |$B_{1}^{+}$| map (see Figure S6), the reconstructed conductivity in the bottom row shows an error, as indicated by the red arrow. This results in a mismatch in *D*_ϕ_, as the conductivity error propagates to the estimated ϕ^+^ map.

**FIGURE 8:**
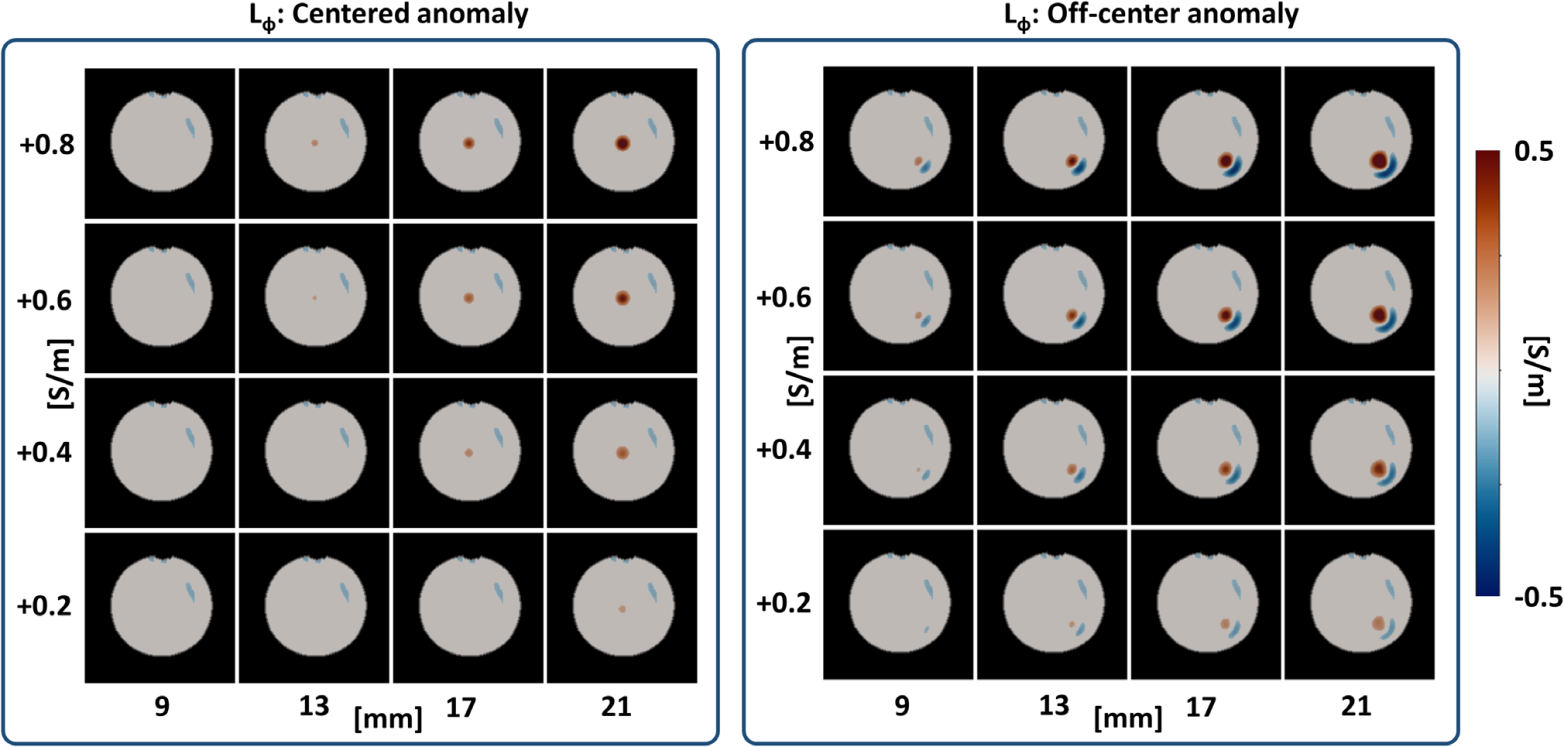
Calculated error maps from $B_{1}^{+}$ prediction to test model sensitivity for varying anomalies in the input conductivity. Anomaly is varied in size (x‐axis), conductivity offset (y‐axis) and location (centered and off‐centered).

Phantom experiment 3: the results from $B_{1}^{+}$ predictions on a spherical phantom with an anomaly are shown in Figure 8. As expected, the model is more sensitive to larger anomalies and anomalies with a higher conductivity offset with respect to the reference conductivity. Furthermore, the sensitivity is dependent on the location of the anomaly. Lower errors are observed with an anomaly in the center (left). This is a consequence of the low phase curvature in the center of the phantom that leads to a reduced sensitivity to distortions introduced by the anomaly.

Therefore, depending on the location of the anomaly, a minimum size and conductivity offset is needed to reliably identify errors. For this simplified case, the model is sensitive for anomalies ≥0.4 S/m and ≥13 mm, regardless of the location of the anomaly. When the anomaly is located in the periphery smaller sizes and lower conductivity offsets can also be detected.

### 4.3 Applicability to in vivo MRI data

**FIGURE 9:**
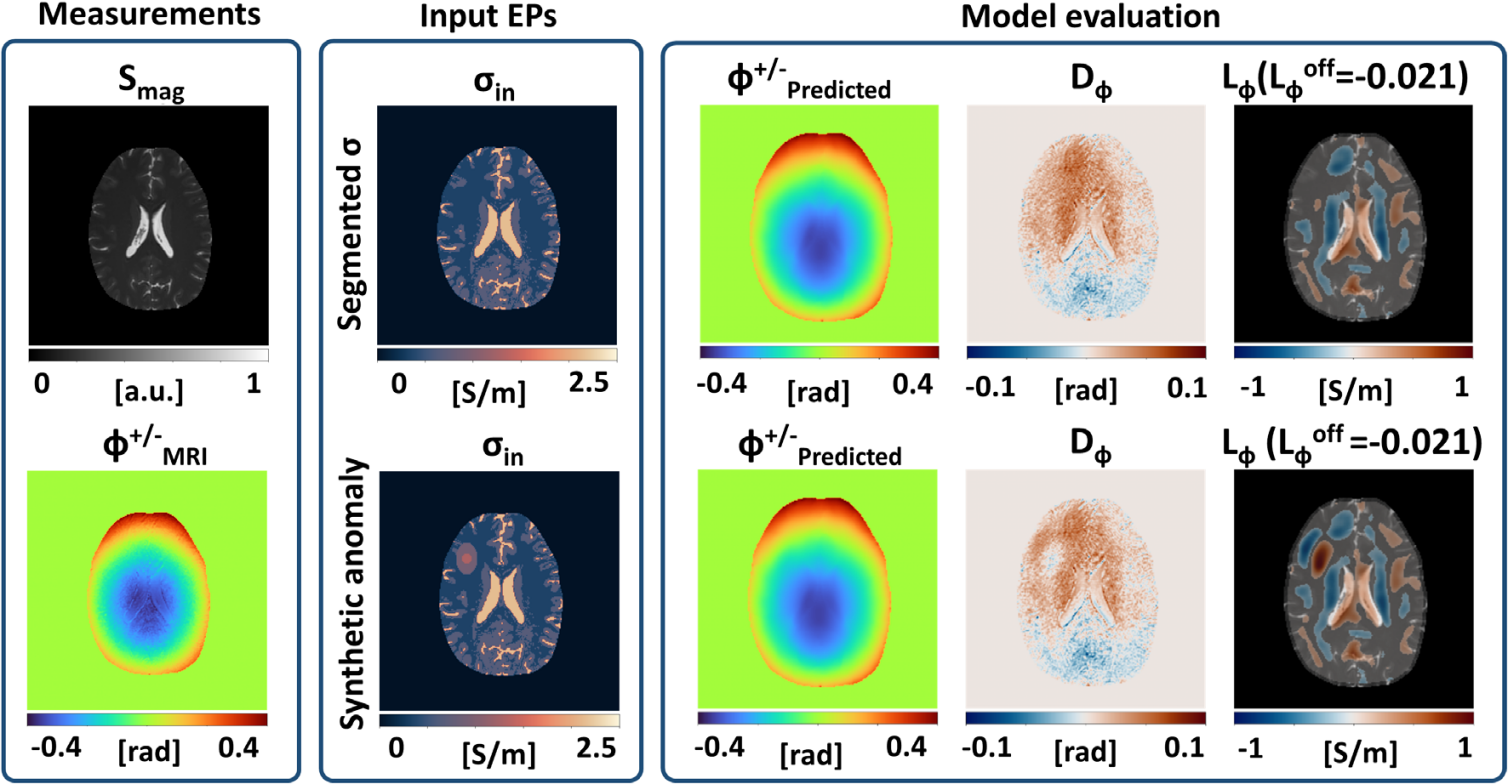
Calculated $B_{1}^{+}$ for segmentation based electrical properties (EPs) in an in vivo brain scan. The first row shows $B_{1}^{+}$ calculation on the segmentation based reconstruction, the second row shows $B_{1}^{+}$ calculation with an added synthetic anomaly. *L*_ϕ_ values are shown for values >0.2 S/m.

In the final experiment, the method was applied to in vivo brain data (Figure 9). The *D*_ϕ_ and *L*_ϕ_ maps show significant errors, higher than what was observed in Figure 3: The MAE = 2.4e−2 and 2.3e−2 rad without (top row) and with the synthetic anomaly (bottom row), respectively. This can be explained by the imperfect EPs values that were created from tissue segmentation. The offset *L*_ϕ_^off^ = −0.02 shows that a small conductivity offset is present with for both cases.

Despite the higher errors overall, the region where a synthetic tumor was added to the input conductivity (left frontal lobe) can clearly be identified from the changes in the *D*_ϕ_ and high errors in the *L*_ϕ_ maps. This shows that errors (false positives) in the reconstructed EPs can be identified for an in vivo case, even in regions with complex geometries such as the brain.

## 5 DISCUSSION

In this work, we presented a method for the evaluation of reconstructed EPs from an arbitrary EPT reconstruction method. This was done using a fast physics‐based model that estimates complex $B_{1}^{+}$ fields from reconstructed EPs, provided with boundary information of the measured $B_{1}^{+}$ fields. By analyzing the difference between the estimated and measured $B_{1}^{+}$ field (*D*_ϕ_ maps), errors with respect to the underlying EPs can be identified in 90 s for a full 3D brain at 1 mm^3^ isotropic resolution. To relate the observed differences to local errors in conductivity, surrogate error maps (*L*_ϕ_) were used. The model was first validated using simulated data and afterward applied to investigate how errors in EPs can be identified with the predicted $B_{1}^{+}$ maps. The method was further tested on MRI measurements, both in phantoms and in vivo to demonstrate the applicability and sensitivity of the approach in realistic settings.

The experiments on the simulated data showed that the proposed method is able to accurately reconstruct $B_{1}^{+}$ fields, which match the GT fields from finite‐difference time‐domain (FDTD) simulation. With the assumption that ∇*B*_*z*_ = 0,^31^ necessary for application in practice, small discrepancies between the predicted and reference $B_{1}^{+}$ map arise. As demonstrated, these discrepancies are small, both in absence and presence of noise. Other assumptions, such as the transceive phase assumption and the homogeneous Neumann boundary condition on the EPs also have minor effect on the estimated $B_{1}^{+}$ maps. Despite these model‐related errors in estimated $B_{1}^{+}$ maps, discrepancies arising from the use of erroneous EPs maps in input can be identified. The simulation results show that offsets of 0.05 S/m (˜10% error in EPs) can be detected (MAE of 2e−2 rad), which is in line with previous work.^41^ As typical reconstruction errors are >10%,^21^ sensitivity is high enough to detect such errors. Additionally, local anomalies can be detected, even in presence of noise.

The experiments with the spherical phantoms show that $B_{1}^{+}$ prediction is also applicable on MRI data and works with actual EPT reconstructions. It is especially effective for the detection and identification of global conductivity offset errors and for the detection of regions larger than 1 cm in diameter that contain incorrect conductivities ≥0.4 S/m with respect to the GT. This 1 cm resolution is in line with the results presented in a recent study.^42^

For in vivo measurements, without any GT EPs available, larger errors were observed. This can be expected because of the more intricate geometry of the brain in comparison to a spherical phantom. This increases reconstruction difficulty as well as the model error of the $B_{1}^{+}$ prediction. However, anomalies in the conductivity can still be identified, even with imperfect EP reconstruction in the rest of the brain. This shows applicability of the method in practice.

Reconstruction evaluation has been used before in the context of polynomial fitting‐based reconstructions, where the discrepancy between the fitted phase and the measured phase is used as reliability measure.^43^, ^44^ However, in contrast to these methods, the proposed method is applicable to any EPT reconstruction and does not make use of the local homogeneity assumption. The capability to quickly evaluate EPT reconstructions makes the proposed method especially useful for DL‐based EPT reconstructions. Such reconstructions often seem visually reasonable, in comparison to for example Helmholtz reconstruction, which are often heavily influenced by noise and boundary artifacts. However, large errors such as conductivity offsets and incorrect pathology reconstruction can still be present because of for example generalization problems.^22^ The black box nature of these reconstructions makes it difficult to identify these errors efficiently. Using predicted $B_{1}^{+}$ fields it is possible to quickly identify potential errors in the reconstruction. This can increase trust in DL‐based reconstructions.

It should be noted that both FDTD simulations (e.g., Sim4Life as used in this work) and the presented method are based on FD calculations. Although FDTD simulations could similarly be used to evaluate EPs reconstructions, they have several disadvantages for evaluation purposes. For example, FDTD simulations require coil details and EP information outside the ROI and can take several hours. Instead, the proposed method only relies on the measured $B_{1}^{+}$ field at the boundary of the ROI and the reconstructed EPs, which simplifies calculations and setup and is much faster to apply. These advantages make it possible to easily adopt the method in a clinical workflow. Contrarily, because of the reliance on $B_{1}^{+}$ field information at the boundary of the ROI, which indirectly contains coil‐specific field information, the proposed method is not a direct substitute for FDTD simulations to simulate fields.

As EPT is a complex 3D problem, the proposed method is formulated in similar fashion. This has several consequences. Although $B_{1}^{+}$ prediction can also be done in 2D, missing the third dimension will deteriorate the accuracy of $B_{1}^{+}$ estimation. Furthermore, in a lot of EPT experiments only the conductivity is estimated. Because the model takes the complex permittivity as input, an estimation of the relative permittivity is also needed. Simplifications to the relative permittivity will reduce the accuracy of $B_{1}^{+}$ prediction, as shown in Figure S7, but errors in the conductivity can still be detected.

Because the proposed method evaluates EPs by solving the forward problem, an obvious next step is using the calculated $B_{1}^{+}$ discrepancies to update EPs, as alternative to previously presented iterative methods.^31^, ^45^, ^46^, ^47^, ^48^ Similarly to the proposed method, the forward models presented in these iterative reconstructions could also be used for evaluation purposes. However, disadvantages, such as model simplifications,^46^ required knowledge of the incident *B* field,^47^, ^48^ and applicability in realistic settings should first be investigated. Additionally, other applications of $B_{1}^{+}$ estimation can be explored in the future. As an example, instead of application to the entire brain region, the model could also be used in a limited region such as a tumor to limit influences of errors outside of this region.

In conclusion, $B_{1}^{+}$ prediction and its use for evaluation can be instrumental for the translation of EPT to a clinical setting. It provides the possibility to evaluate EP reconstructions in vivo and, therefore, to be used as a confidence indicator for the reconstructed EP maps, since no GT EPs are otherwise available. This is particularly relevant for DL‐based reconstructions in vivo, where generalization to unseen structures remains an issue.

## 6 CONCLUSION

In this work, we have presented a method to predict complex $B_{1}^{+}$ fields from EPs maps reconstructed with MR‐EPT methods. The physics‐based model reconstructs $B_{1}^{+}$ maps in 90 s for a realistic brain size and is easily applicable in vivo without necessary a‐priori information such as coil geometry. The method was used to evaluate EPs estimated with EPT.

Evaluation of EPs using this predicted $B_{1}^{+}$ map is especially effective to detect erroneous anomaly predictions and at detecting global offsets in conductivity input. The method is, therefore, suitable to evaluate and provide confidence for in vivo EPT reconstructions. As a result, it can be an important tool for clinical translation of EPT in the future.

## FUNDING INFORMATION

Netherlands Organization for Scientific Research (NWO), VENI Grant number: 18078.

## CONFLICT OF INTEREST STATEMENT

The authors declare no potential conflict of interests.

## Supporting information


**Figure S1.** Analysis of the transceive phase error made with the $B_{1}^{+}$ estimation in comparison to the simulated transceive phase error (A). In the blue square (B) the boundary condition of the transmit phase was used, in the green square (C and D) the boundary condition of the transceive phase was used. Option C is used in practice in the paper.
**Figure S2.** Coronal orientation of $B_{1}^{+}$ prediction using the model from Equation [5] and ground truth electrical properties as input.
**Figure S3.** Sagittal orientation of $B_{1}^{+}$ prediction using the model from Equation [5] and ground truth electrical properties as input.
**Figure S4.** Coronal orientation of $B_{1}^{+}$ prediction using the model with assumptions (Equation [6]) and ground truth electrical properties as input.
**Figure S5.** Sagittal orientation of $B_{1}^{+}$ prediction using the model with assumptions (Equation [6]) and ground truth electrical properties as input.
**Figure S6.** Measured |$B_{1}^{+}$| of the sphere phantom 3 (σ = 0.61 S/m), containing an artifact on the right side of the phantom. This artifact causes a reconstruction error in the conductivity, as shown in Figure 7.
**Figure S7.** $B_{1}^{+}$ prediction for combinations of correct and modified EPs. In A, the modified conductivity is 10% higher, with constant permittivity. In *B*, the modified EPs have a constant value of σ = 0.7 S/m and ε_*r*_ = 65. From the predicted phase map, *D*_ϕ_ and *L*_ϕ_ are shown.

## A DERIVATION OF THE RECURRENT RELATIONS

To solve the partial differential equation in Eq. [3] for $B_{1}^{+}$, first and second order (central) FDs can be used to approximate the partial derivatives, defined in 1D as:

$$\frac{\partial }{\partial x}f\left(x\right)=\frac{f\left(x+h\right)-f\left(x-h\right)}{2h}$$

(A1)
$$\begin{array}{ll} \frac{\partial^{2}}{\partial x^{2}}f\left(x\right) & =\frac{f\left(x+h\right)-2f\left(x\right)+f\left(x-h\right)}{h^{2}}\\ & =\frac{f\left(x+h\right)+f\left(x-h\right)}{h^{2}}-\frac{2f\left(x\right)}{h^{2}}, \end{array}$$

with *h* the resolution. For a region of interest of *N* voxels (where *N* is equivalent to the size of the domain Ω), these spatial derivatives can be written as sparse matrix operators [*N* × *N*]^30^:

$$\frac{\partial }{\partial x}=D_{x}=\frac{1}{2h}\left[\begin{array}{llllll} -\;1 & 0 & 1 & & & \\ & -1 & 0 & 1 & & \\ & & \ddots & \ddots & \ddots & \\ & & & -\;1 & 0 & 1 \end{array}\right],$$

(A2)
$$\begin{array}{cc} \frac{\partial^{2}}{\partial x^{2}} & =L_{x}=\frac{1}{h^{2}}\;\left[\begin{array}{cccccc} 1 & 0 & 1 & & & \\ & 1 & 0 & 1 & & \\ & & \ddots & \ddots & \ddots & \\ & & & 1 & 0 & 1 \end{array}\right]\\ & \;+\frac{1}{h^{2}}\left[\begin{array}{cccccc} 0 & -2 & 0 & & & \\ & 0 & -2 & 0 & & \\ & & \ddots & \ddots & \ddots & \\ & & & 0 & -2 & 0 \end{array}\right]. \end{array}$$

Where *D*_*y,z*_/*L*_*y,z*_ have similar shape. The spatial derivatives of a discrete $B_{1}^{+}$ field can be written as matrix vector product where the FD derivative operators from Eq. [A2]] act on all *N* voxels in the region of interest:

$$\frac{\partial }{\partial x}B_{1}^{+}=\frac{D_{x}B}{h}$$

(A3)
$$\frac{\partial^{2}}{\partial x^{2}}B_{1}^{+}=\frac{L_{x}B}{h^{2}}-\frac{2B}{h^{2}}.$$

Here, *B* [*N* × 1] represents the $B_{1}^{+}$ field. Generalizing the 1D FD operators in Eq. [A3] to 3D, Eq. [1] can be rewritten as a matrix equation with FDs expressing the spatial derivatives as:

(A4)
$$\begin{array}{ll} & -\mathrm{LB}+6B=\omega^{2}\mu_{0}\mathrm{EB}h^{2}\\ & \;-\left(D_{x}\ln E+iD_{y}\ln E\right)\left(D_{x}B-iD_{y}B+\frac{1}{2}D_{z}Z\right)\\ & \;-D_{z}\ln E\left(D_{z}B-\frac{1}{2}D_{x}Z-\frac{i}{2}D_{y}Z\right). \end{array}$$

Here, *L* is the Laplacian operator in 3D (*L*_*x*_ + *L*_*y*_ + *L*_*z*_). *E* [*N* × 1] is the complex permittivity, obtained via an arbitrary EPT reconstruction method, and represents the input to the model. *Z* [*N* × 1] represents *B*_*z*_.

Equation [A4] can be rewritten to isolate the terms without a derivative operator acting on it:

(A5)
$$\begin{array}{ll} & \left(6-\omega^{2}\mu_{0}Eh^{2}\right)B=\mathrm{LB}\\ & \;-\left(\left(D_{x}\ln E+iD_{y}\ln E\right)\left(D_{x}-iD_{y}\right)+D_{z}\ln ED_{z}\right)B\\ & \;+\frac{1}{2}\left(-\left(D_{x}\ln E+iD_{y}\ln E\right)D_{z}+D_{z}\ln E\left(D_{x}+iD_{y}\right)\right)Z. \end{array}$$

When considering *E* known, all operators acting on *B* and *Z* can be combined into two operators (here called *A* and *A*_*z*_), only acting on off‐diagonal terms of *B* and *Z*. Using this, *B* in a voxel is expressed in terms of *B* and *Z* in neighboring voxels through a recurrent relation^29^:

(A6)
$$B_{i+1}=\frac{AB_{i}+A_{z}Z}{6-\omega^{2}\mu_{0}Eh^{2}},$$

where *i* denotes the recurrent application number.
