# Supplementary material for: Electrical properties based B1+ prediction for electrical properties tomography reconstruction evaluation
Source: Magn Reson Med. 2025 Apr 2;94(3):1269–83. doi: 10.1002/mrm.30520 (PMC12202738; doi:10.1002/mrm.30520)
Supplement: Supplementary file 1 — Figure S1. Analysis of the transceive phase error made with the B1+ estimation in comparison to the simulated transceive phase error (A). In the blue square (B) the boundary condition of the transmit phase was used, in the green square (C and D) the boundary condition of the transceive phase was used. Option C is used in practice in the paper. Figure S2. Coronal orientation of B1+ prediction using the model from Equation [5] and ground truth electrical properties as input. Figure S3. Sagittal orientation of B1+ prediction using the model from Equation [5] and ground truth electrical properties as input. Figure S4. Coronal orientation of B1+ prediction using the model with assumptions (Equation [6]) and ground truth electrical properties as input. Figure S5. Sagittal orientation of B1+ prediction using the model with assumptions (Equation [6]) and ground truth electrical properties as input. Figure S6. Measured |B1+| of the sphere phantom 3 (σ = 0.61 S/m), containing an artifact on the right side of the phantom. This artifact causes a reconstruction error in the conductivity, as shown in Figure 7. Figure S7. B1+ prediction for combinations of correct and modified EPs. In A, the modified conductivity is 10% higher, with constant permittivity. In B, the modified EPs have a constant value of σ = 0.7 S/m and ε r = 65. From the predicted phase map, D ϕ and L ϕ are shown. [file MRM-94-1269-s001.docx]

Supporting information

**Electrical Properties based B_1_^+^ prediction for Electrical Properties Tomography reconstruction evaluation**

T.G. Meerbothe, K.J Jung, C. Cui, D.H. Kim, C.A.T. van den Berg, S. Mandija

**Supporting information 1: Calculation and analysis of the estimation of the transceive phase.**

The transceive phase is a combination of the transmit phase and the receive phase, defined as:

$$\phi^{\pm}=\phi^{+}+\phi^{-}$$

To estimate this transceive phase, a combination of the estimated transmit phase (as described in section 2.2) and an estimated receive phase (which can be estimated in similar fashion) can be used. The complex receive field $B_{1}^{-}=|B_{1}^{-}|e^{-i\phi^{-}}$ is similarly related to the EPs as the complex transmit B_1_^+^ in section 2.1:

$$-\nabla^{2}B_{1}^{-}=\omega^{2}\mu_{0}\varepsilon_{c}B_{1}^{-}-\left( \frac{\partial B_{1}^{-}}{\partial x}+i\frac{\partial B_{1}^{-}}{\partial y} \right)\left( g_{x}-ig_{y} \right)-\frac{\partial B_{1}^{-}}{\partial z}g_{z}$$

Thus, the B_1_^-^ field can thus be similarly approximated using a recurrent relation:

$$B_{i+1}^{-}=\frac{{A_{-}B}_{i+1}^{-}}{6-{\mu_{0}\omega}^{2}Eh^{2}}$$

Where *B^-^* here depicts B_1_^-^, and *A_-_* represents the corresponding operator on B_1_^-^. The transceive phase can now be approximated using a combination of B_1_^+^ and B_1_^-^  as:

$$\hat{\phi^{\pm}}=\angle(\hat{B_{1}^{+}}\left( \varepsilon_{r} \right))+\angle(\hat{B_{1}^{-}}\left( \varepsilon_{r} \right))$$

The main problem with this approach is that only |B_1_^+^| and ф^±^ are measured in practice, and thus available as boundary conditions. For the prediction of the transceive phase |B_1_^-^| is however also necessary. An approximation can be done by using |B_1_^+^| as boundary condition for both B_1_^+^ and B_1_^-^. This approach has been tested and is shown in figure S1.

When the transmit phase is directly compared to the transceive phase, a transceive phase error is made. For the simulated brain model, also used in the simulation experiments, this transceive phase error is shown in figure S1 A. The model error, when comparing the predicted transmit phase with the simulated ground truth, using the correct boundary conditions, is shown in B. This error differs significantly from the transceive phase error and has lower errors overall. Estimations in realistic scenarios, using |B_1_^+^| and ф^±^ as boundary condition, are shown in C (for an estimated transmit phase) and D (for an estimated transceive phase). These estimations both show similar errors in comparison to the ideal case presented in B, and again lower errors in comparison to the transceive phase error. Additionally, predicting the transceive phase (D) instead of the transmit phase (C) only marginally improves the estimation. Therefore, the method in C, where the transmit phase is predicted, with transceive phase boundary conditions, is used for MRI measurements in this paper.


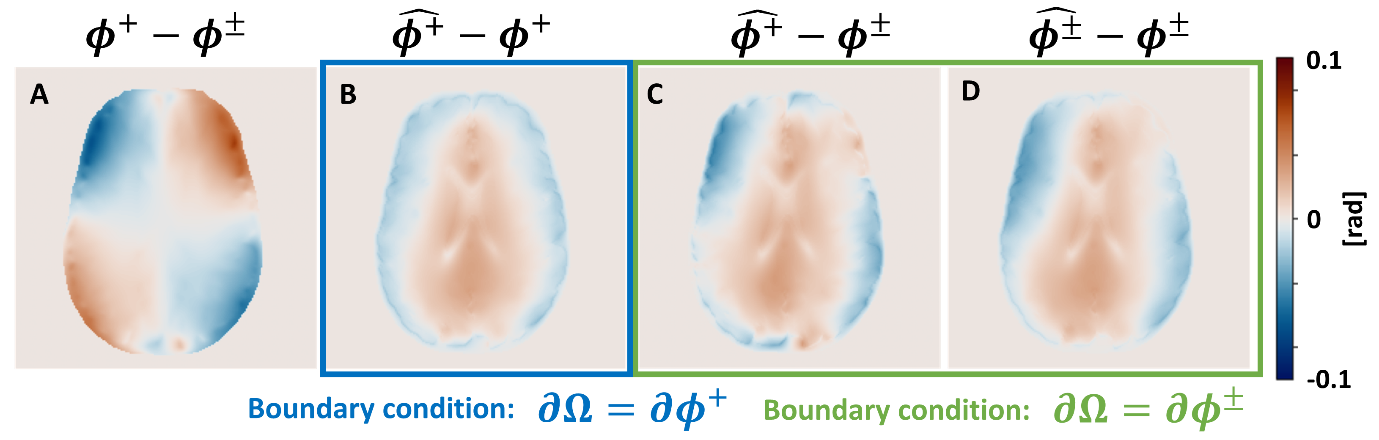


**Figure S1:** Analysis of the transceive phase error made with the B_1_^+^ estimation in comparison to the simulated transceive phase error (A). In the blue square (B) the boundary condition of the transmit phase was used. In the green rectangle (C and D) the boundary condition of the transceive phase was used. Option C is used in practice in the paper.

**Supporting information 2: Different orientations of the predicted B_1_^+^ field when including B_z_ information.**

In addition to the axial orientation, coronal and sagittal orientations for the B_1_^+^ prediction with inclusion of B_z_ information are shown in figure S2 and figure S3. Similarly as for the axial slice, reconstructed differences in other orientations show negligible values, indicating the validity of the used model and implementation.

**
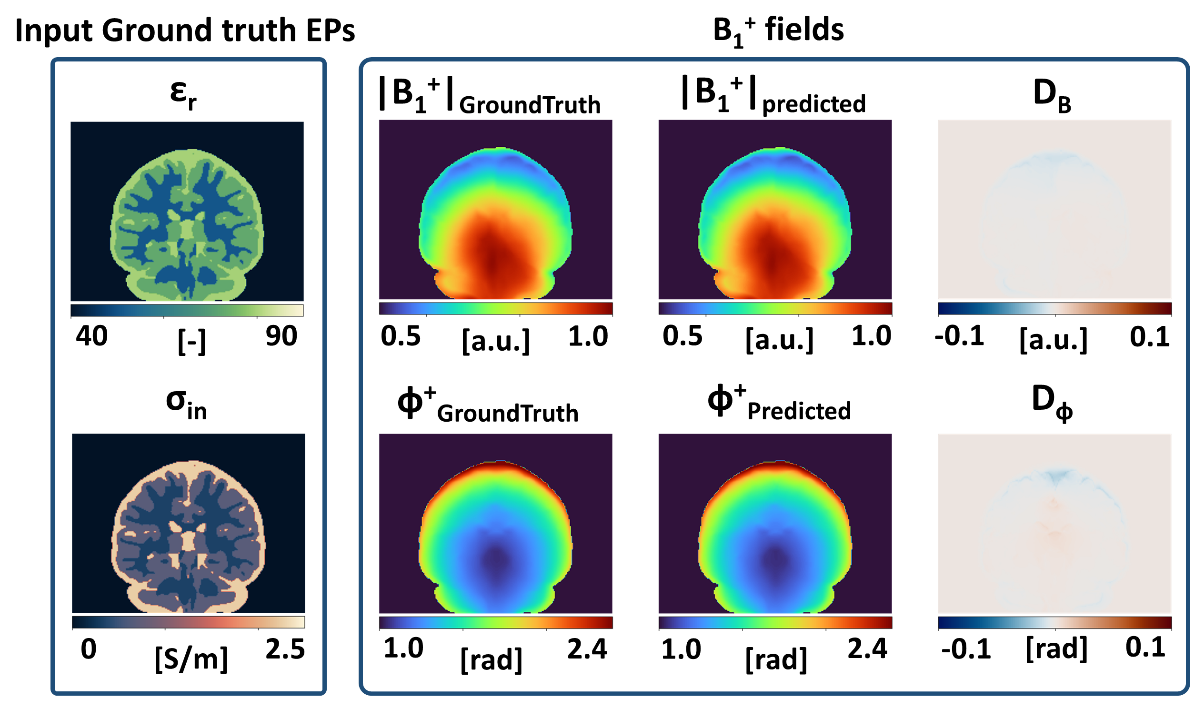
**

**Figure S2:** Coronal orientation of B_1_^+^ prediction using the model from eq. 5 and ground truth electrical properties as input.

**
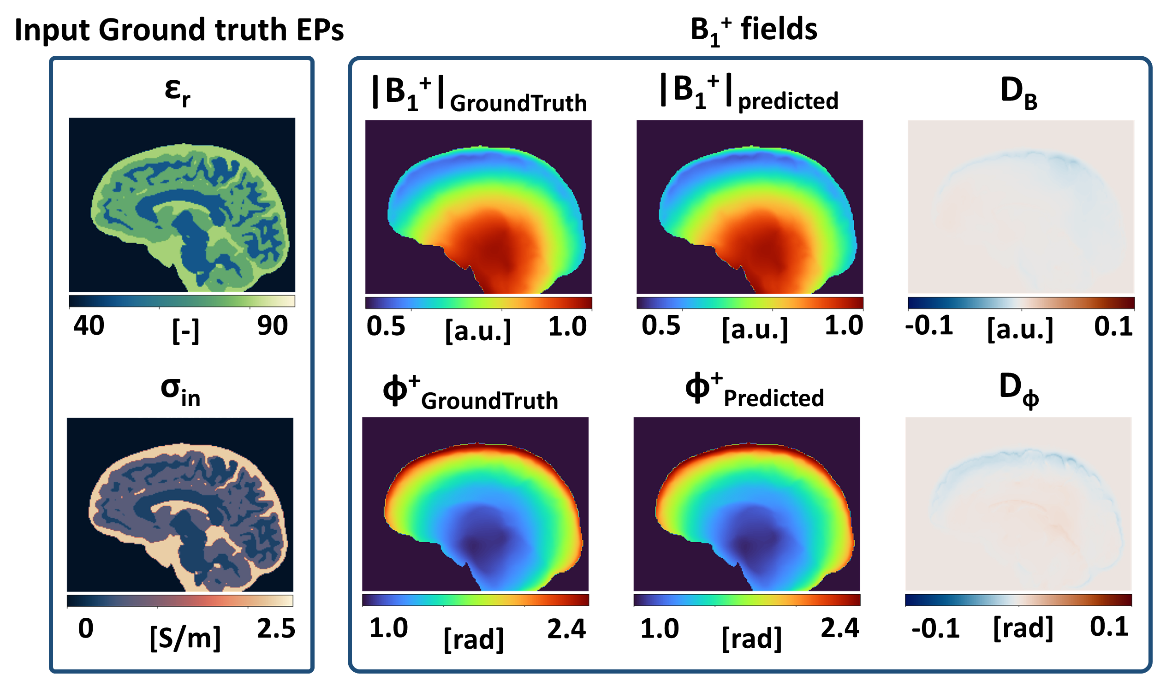
**

**Figure S3:** Sagittal orientation of B_1_^+^ prediction using the model from eq. 5 and ground truth electrical properties as input.

**Supporting information 3: Different orientations of the predicted B_1_^+^ field when excluding B_z_ information.**

Coronal and sagittal orientations for the B_1_^+^ prediction without B_z_ information is shown in figure S4 and S5 in a noisy (SNR=40) case. Similarly as for the axial slice, reconstructed differences are higher in comparison to the full model, as can be expected. Especially in the top of the brain higher differences are present.

**
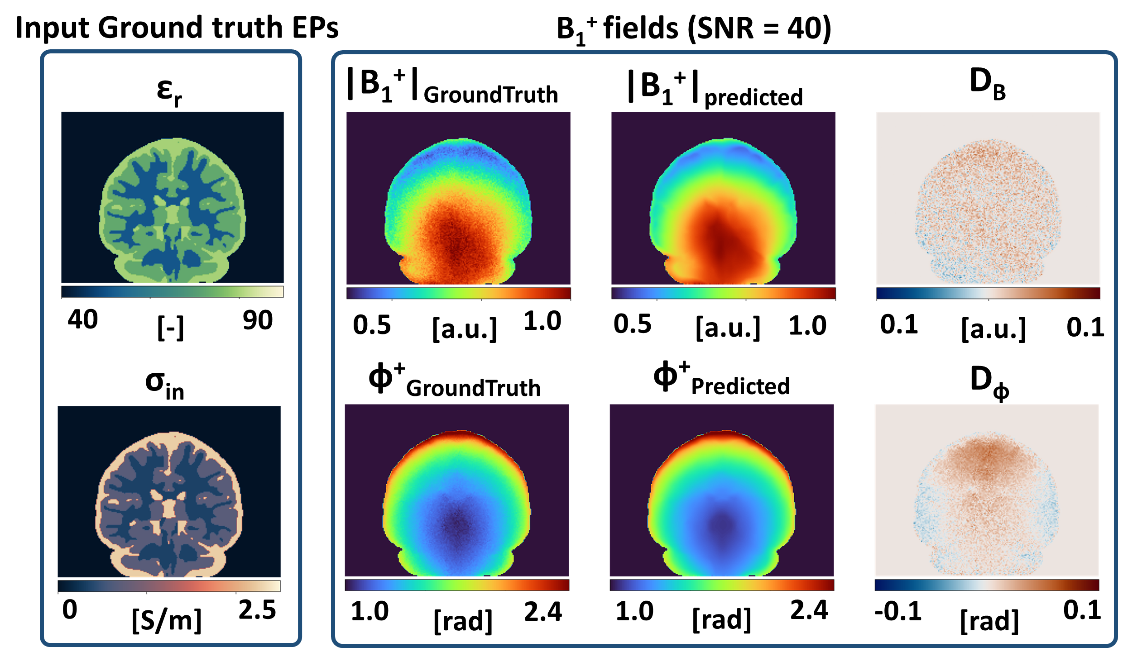
**

**Figure S4:** Coronal orientation of B_1_^+^ prediction using the model with assumptions (eq. 6) and ground truth electrical properties as input.

**
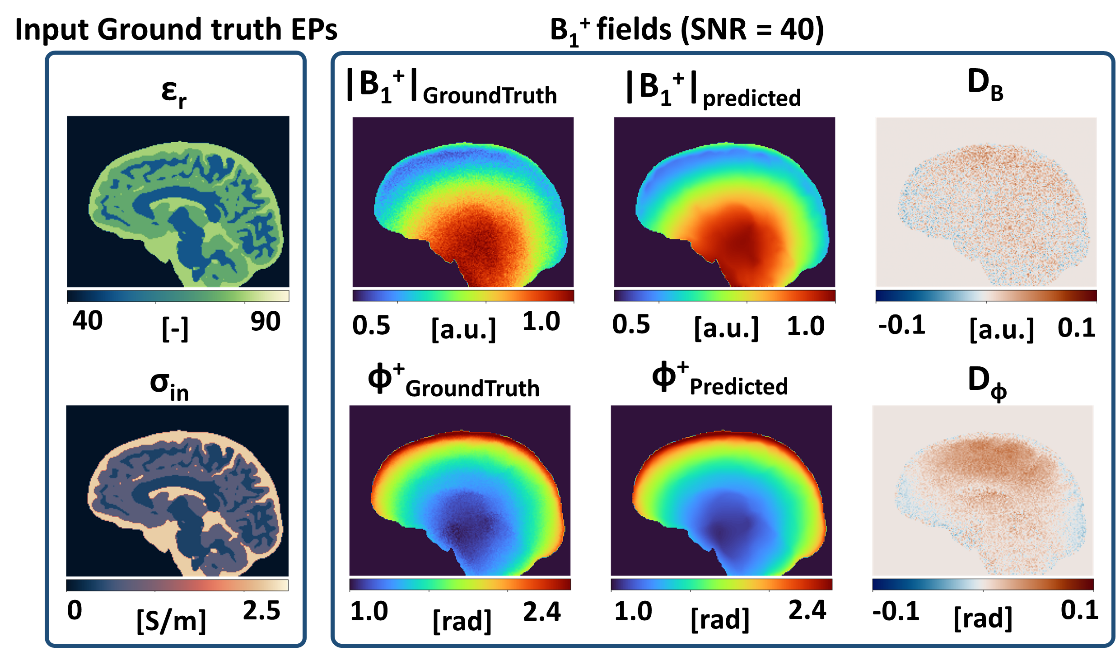
**

**Figure S5:** Sagittal orientation of B_1_^+^ prediction using the model with assumptions (eq. 6) and ground truth electrical properties as input.

**Supporting information 4: Measured |B_1_^+^| of sphere with σ=0.61 S/m.**

The artifact in the complex Helmholtz reconstruction presented in figure 7 is due to an imaging artifact in the measured |B_1_^+^| data. The measured |B_1_^+^| data is shown below in figure S6.

**
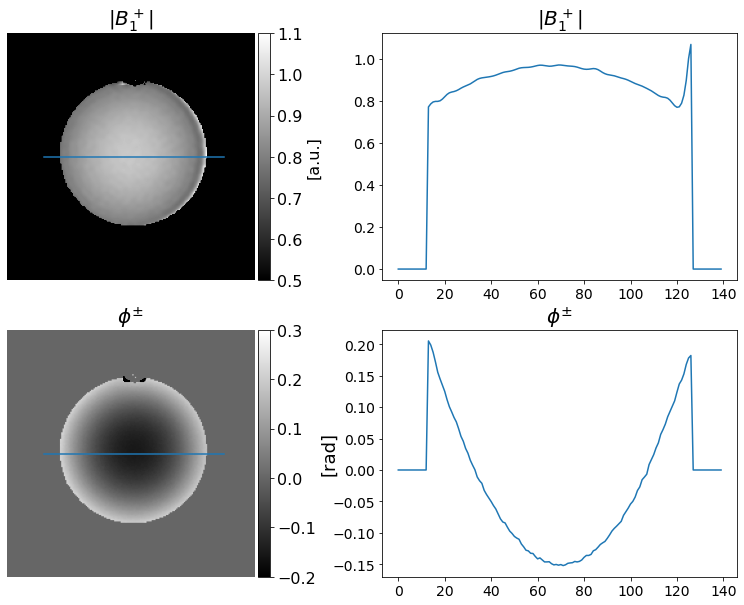
**

**Figure S6:** Measured |B_1_^+^| of the sphere phantom 3 (σ=0.61 S/m), containing an artifact on the right side of the phantom. This artifact causes a reconstruction error in the conductivity, as shown in figure 7.

**Supporting information 5: Influence of permittivity on the resulting phase map**

In a large part of this work, only changes in input conductivity are evaluated using the predicted phase map. However, the permittivity does also influence the phase in practice. To judge the influence of the conductivity and permittivity individually on the estimated phase map, two experiments were done with the two compartment spherical phantom and the simulated brain phantom without tumor inclusion described in section 3.1. In both experiments, the complex B_1_^+^ map was estimated four times using:

1. Ground truth EPs
2. Ground truth σ and homogeneous permittivity (ε_r_=65)
3. 10% higher σ than the ground truth and ground truth ε_r_
4. 10% higher σ than the ground truth and homogeneous permittivity (ε_r_=65)

The homogeneous permittivity represents the case where permittivity was not reconstructed and is instead assigned a homogeneous value. The conductivity error represents an overprediction error as could be present with phase based EPT.

In the first experiment, (figure S7A) this is shown for the sphere phantom. In the second experiment (figure S7B) the same is done for the brain phantom.

From figure S7 can be seen that incorrect EPs result in different errors in the predicted phase maps. As expected from literature, an error in the permittivity causes a smaller error in the predicted phase in comparison to an error in the conductivity. This is the case for both the sphere and the brain phantom. The L_φ_ map similarly shows that the permittivity indeed has a smaller effect on the predicted phase. Consequently, conductivity can still be evaluated using the phase map, even when incorrect permittivity values are used input.


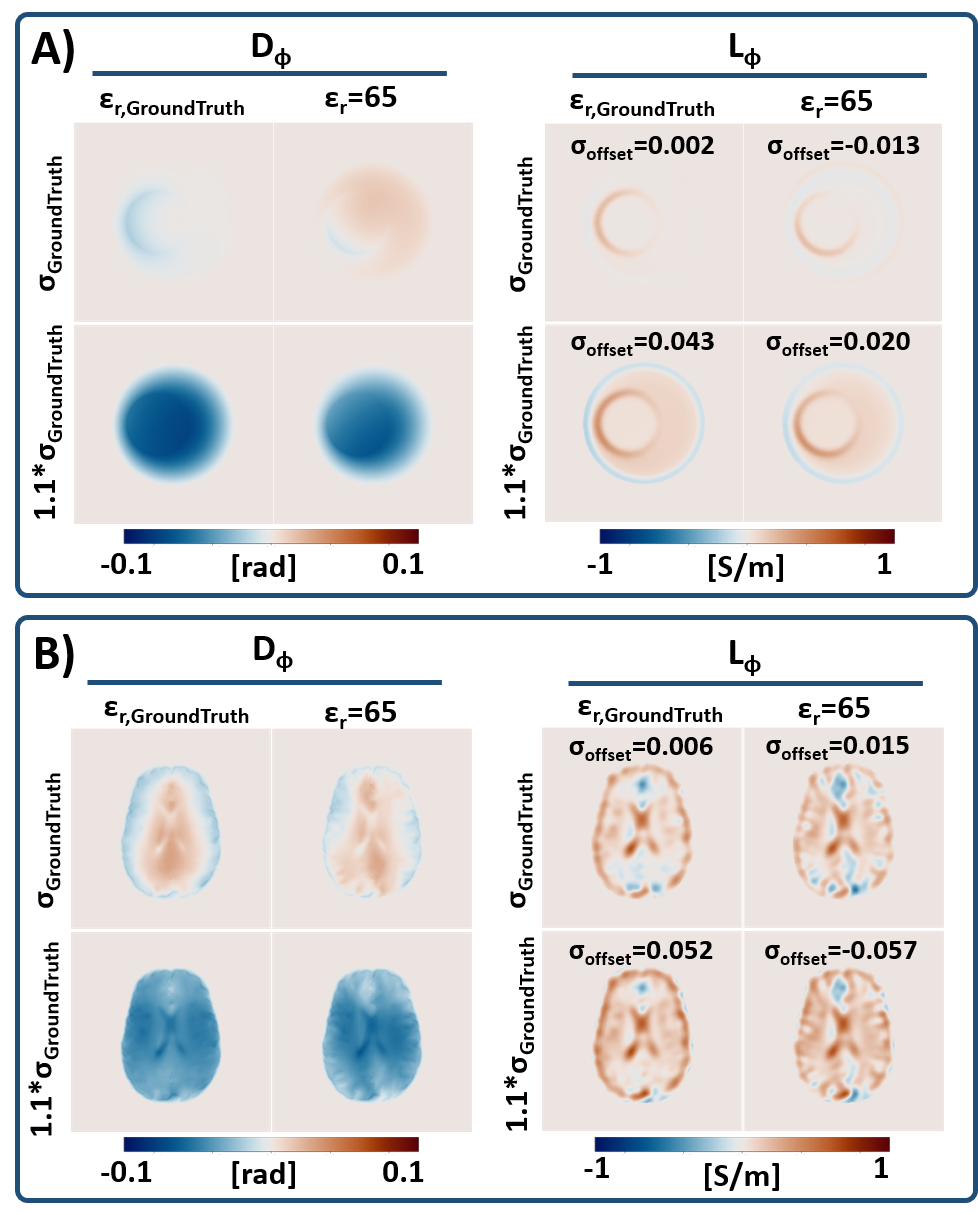


**Figure S7:** B_1_^+^ prediction for combinations of correct and modified EPs. In A), the modified conductivity is 10% higher, with constant permittivity . In B), the modified EPs have a constant value of σ=0.7 S/m and ε_r_=65. From the predicted phase map, D_φ_ and L_φ_ are shown.
